# Bioadhesion in ascidians: a developmental and functional genomics perspective

**DOI:** 10.1098/rsfs.2014.0061

**Published:** 2015-02-06

**Authors:** Roberta Pennati, Ute Rothbächer

**Affiliations:** 1 Dipartimento di Biologia, Università degli Studi di Milano, Milan, Italy; 2 Department of Evolution and Developmental Biology, Zoological Institute, University Innsbruck, Innsbruck, Austria

**Keywords:** bioadhesion, ascidians, papillae, functional genomics, gene networks

## Abstract

The development of bioadhesives inspired from marine animals is a promising approach to generate new tissue-compatible medical components. A number of marine species, through their adhesive properties, also represent significant foulers that become increasingly problematic to aquaculture, shipping or local biodiversity. In order to develop more sophisticated man-made glues and/or efficient fouling resistant surfaces, it is important to understand the mechanical, structural and molecular properties of adhesive organs in selected species. Ascidians are marine invertebrates with larvae that opportunistically attach to almost any type of submerged surface to undergo metamorphosis into permanently sessile adults. Not only do they represent a globally important fouling organism, but they are becoming increasingly popular as model organisms for developmental biology. The latter is due to their phylogenetic position as the sister group to the vertebrates and their cellular and molecular accessibility for experimentation. In this paper, we review the mechanisms of larval adhesion in ascidians and draw conclusions from comparative analyses of selected species. We further discuss how knowledge from a developmental and functional genomics point of view can advance our understanding of cellular and molecular signatures and their hierarchical usage in animal adhesive organs.

## 1. Introduction

The mechanisms of temporary and permanent adhesion by marine organisms, termed bioadhesion, present a wealth of novel materials and may guide the design of biomimetic adhesives [1]. Adhesives employed by marine organisms could be particularly useful in the medical field because they can cure (harden) in wet environments and are likely to be tissue compatible. What is more, an improved understanding of bioadhesion, particularly by problematic biofouling organisms, could assist in developing environmentally friendly marine antifouling formulations [2].

Sessile marine organisms have developed a wide variety of multicomponent bioadhesives to survive in unpredictable environmental conditions [3,4]. The large variety of fouling organisms, with similarly diverse adhesive strategies and remarkably complex adhesive components, have made it difficult to find a universal antifouling solution but offer great potential for biomimetic materials.

Generally, animal adhesive secretions are based on proteins [5], but these proteins can vary widely between organisms. Mussel byssus and barnacle cement, perhaps the most studied adhesive secretions, contain very different adhesive proteins. Adult mussels bind to submerged surfaces by secreting a bundle of threads, known as byssus from glands located in the mussel foot [6]. So far, five mussel foot proteins have been identified in the byssus. Notably the first two proteins to be secreted on the substrate surface (Mfp/3 and Mfp/5) have a high content of the catecholic amino acid 3,4-dihydroxyphenyl-l-alanine (DOPA) [7]. Adhesion to the substrate and cohesion between Mfps is achieved by a fine-tuned redox control mechanism that acts to cross-link DOPA residues [8]. Indeed, in biomimetic engineering, catechol moieties that mimic mussel adhesive proteins are now being functionalized with synthetic polymers to provide diverse adhesive, sealant and coating properties, notably for biomedical applications [9]. In contrast with mussels, barnacle cement proteins do not appear to require any post-translational modifications, particulary DOPA. This suggests a completely different adhesive strategy from that employed by mussels, although further clarification is required [10,11].

Ascidians (sea squirts) are one group of marine organisms that are particularly interesting in this context of bioadhesion. Not only are ascidians important foulers [12], they also have easily accessible larvae that can be observed in the laboratory for adhesive settlement properties, for stepwise building of their adhesive organs and at a cellular and molecular level. Indeed, ascidians include several well-developed model organisms with vast repositories of genomic and bioinformatic data linked to phenotypes that can be explored *in silico* prior to experimental set-up [13]. Worldwide, ascidians are among the most prominent fouling threats to aquaculture, primarly due to a rapid growth rate combined with the ability to settle on a wide range of substrata [14]. To prevent ascidian fouling, anti-metamorphic properties of allelochemicals [15] or neurotransmitters [16] may be considered.

This review summarizes the ascidian system in light of bioadhesion research. Surprisingly little is known about the exact adhesive strategies of ascidians. While the larval adhesive organs, named palps or papillae, were described morphologically [17,18], the exact number, nature and combination of glue-forming cells, including so-called collocytes, seems less clear (see below). Furthermore, their composite content and the adhesion-producing mechanism remain largely elusive. Interestingly, ascidians produce post-translational modifications forming DOPA and TOPA (3,4,5-trihydroxyphenylalanine) in their blood cells to bind metal ions and vanadium [19] but a link to adhesion was never made. What is more, putative DOPA-forming enzymes or their activity could not be localized to ascidian adhesive organs [20–22]. Proteins rather containing sulfydryl groups were detected in adhesive granules of selected species and a phenol-tanning mechanism was considered unlikely [23].

Approaching a functional signature for wet adhesion in ascidians thus requires deeper knowledge of the larval adhesive organs and the process of adhesion itself, in both mechanistic and molecular terms. The functional units of adhesive production are usually specialized cells triggered to release adhesives from vesicular contents, either at once or in sequence to form an adhesive product strong enough for attachment of the entire organism. The structural and molecular characteristics of these cells within organs will be discussed in a comparative way, and in light of their developmental and evolutionary history. Furthermore, functional genomics tools in ascidians are summarized that will aid in the discovery of further cellular and molecular adhesive signatures.

## 2. Diversity of adhesive organs and attachment in ascidians

Ascidians possess free-swimming larvae, the dispersal stage of their life cycle. After hatching, the larva first swims upwards towards light, with subsequent behavioural changes leading to the larva searching downwards for a shadowed substrate upon which to settle. It explores the substrate for a short period of time by quickly touching it with its adhesive papillae or palps. These are three organs projecting from the anterior epidermis and allow temporary attachment of the larva via mucus secretion. Papillae therefore have both sensory and secretory function. When the larva finds a suitable substrate, it starts metamorphosis, retracts the tail and develops ampullae, the definitive attachment organs, and transforms into a sessile juvenile.

In this section, we will describe and compare the adhesive organs of representative species, allowing unifying conclusions to be drawn. It will be shown that, while the relative arrangement of sensory and secretory cells may vary greatly, two types of neurons are the rule: immediately exposed central neurons and others more basal. These cell types are likely specialized in the tasks of chemo- and mechanosensation, paralleled by secretion from the collocytes for substrate adhesion. Together, they orchestrate larval attachment and metamorphosis.

Adult ascidians can be solitary, such as *Phallusia mammillata* or *Ciona intestinalis*, and usually have simple lecithotrophic tadpole larvae with trunk, locomotory tail and rudimental adult organs. Ascidians can also be compound (colonial), such as *Diplosoma listerianum*, with colonies formed by several tiny individuals enveloped by a common tunic. Compound ascidians usually have more complex larvae, in which several adult organs are already well developed. In any case, all ascidian larvae, with a few exceptions (for instance members of the Molgulidae family), bear two or three adhesive papillae [17,24–26].

Generally, all adhesive papillae are composed of elongated mucus-secreting cells, called collocytes, primary sensory neurons and axial columnar cells. It has been proposed that sensory cells in the palps are mechanosensory neurons playing an important role in the selection of a suitable substrate [18,27,28].

Indeed, the morphology of these organs is very variable among species and they can be classified into 10 types according to their histological characters [29]. A main distinction can be traced between simple conic papillae characteristic of most solitary ascidians, and eversible papillae commonly seen in compound species, which are capable of rapid eversion to expose sticky mucus.

**Figure 1.**
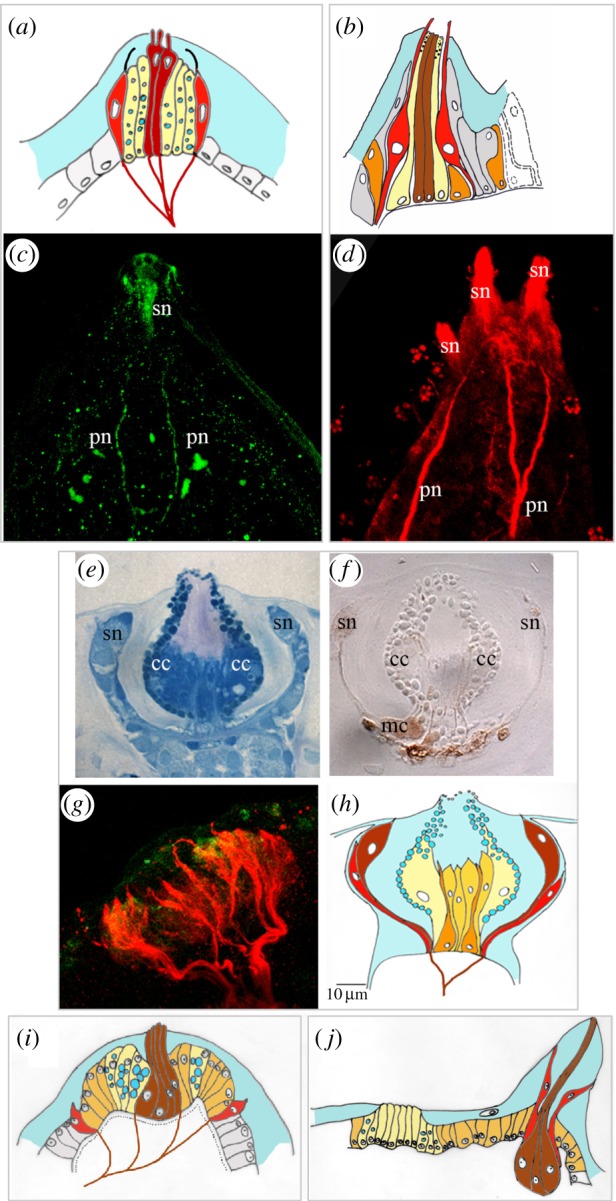
Adhesive papillae in ascidian larvae. (*a*,*b*) Schematic drawings of one papilla of (*a*) *P. mammillata* and (*b*) *C. intestinalis* (adapted from [28]), different colours depict different cell types. Colour code: light yellow: collocytes; brown: axial columnar cells or exposed sensory neurons (see text for explanation); red: lower, ciliated sensory neurons; orange: myoepithelial cells; dark yellow: supporting undifferentiated cells; grey or white: surrounding columnar cells; grey frames group images from one species. (*c*,*d*) Confocal laser images of the adhesive papillae of *P. mammillata* (*c*) and *C. intestinalis* (*d*) immunolabelled with an anti β-tubulin antibody. pn, papillary nerves; sn, sensory neurons. (*e*,*f*) Histological sections of *D. listerianum* adhesive papillae after staining with methylene blue (*e*) and after histochemical reaction for detecting acetylcholinesterase activity (*f*). cc, collocyte; mc, myoepithelia cells; sn, sensory neurons. (*g*) Confocal laser microscopy image of an adhesive papilla of *D. listerianum* double immunolabelled with an anti-tubulin antibody (red signal) and anti-serotonin antibody (green signal). (*h*–*j*) Schematic drawings of one papilla of *D. listerianum* (*h*), *C. lepadiformis* (*i*) (modified from [31]) and *B. leachi* (*j*) (modified from [32]). Colour code is the same as in (*a*,*b*).

A *P. mammillata* larva has three simple coniform adhesive papillae positioned at the vertices of a triangular field of two dorsal and one ventral papilla. The larva is completely covered by two layers of the tunic: an inner and an outer one. The tunic is broken at the very tip of each palp and multiple microvilli with bulbous terminations emerge from the central fenestration [30]. By electron microscopy analysis, Dolcemascolo *et al*. [27] found that the central body of the palps is formed by axial columnar cells, characterized by the presence of long microvilli, containing a nucleus at the basal side and large granules in their cytoplasm (figure 1*a*). Immunostaining of the larval nervous system with monoclonal anti neural β-tubulin antibody shows the primary sensory neurons in the palps (figure 1*c*). Fibres from peripheral and central cells join together to form a papillary nerve that enters the central nervous system at the level of the posterior sensory vesicle. The region between the axial columnar cells and the sensory neurons is occupied by elongated mucus-secreting cells (figure 1*a*). The cellular composition of *C. intestinalis* palps is similar to that of *P. mammillata*. The described cell types are all present in *C. intestinalis:* axial columnar cells, collocytes and sensory cells [27] (figure 1*b*). Numerous primary neurons, immunolabelled with anti-tubulin antibodies, join their axons to form papillary nerves (figure 1*d*). Unlike *P. mammillata*, the axial columnar cells of *C. intestinalis* show big digitiform protrusions that pass through the two tunic layers lacking microvilli in their apical part [27].

*Diplosoma listerianum* possess complex everting papillae with axial protrusions named shipate papillae (figure 1*e*–*h*). Each papilla forms a double-walled structure with a central mass of columnar cells (the axial protrusions), an inner wall composed of secretory cells and several myoepithelial cells. These contractile cells can be stained by a histochemical reaction to reveal strong acetylcholinesterase activity and are responsible for rapid eversion of the papillae upon substrate contact (figure 1*f*). Around the rim of the cup-shaped papilla, there are several uniciliated, so-called anchor cells. These are primary sensory cells believed to be mechano- or chemoreceptors and can be immunolabelled with the anti β-tubulin antibody. Axons coming from the anchor cells join to form the papillary nerve (figure 1*g*, red). A very faint signal of serotonin is present in some cells slightly lower than the anchor cells (figure 1*g*, green). The cellular composition of the papillae of *D. listerianum* is very similar to that of *Diplosoma macdonaldi* [33]. Furthermore, in this species, the presence of two kinds of sensory cells was reported: higher anchor cells and lower ciliated neurons whose cilia do not reach the tunic.

Likewise, adhesive papillae of *Clavelina lepadiformis* contain three cell types. Axial columnar cells are localized in the central portion of the papilla and have a fusiform elongated shape with nuclei in the lower third of the cell. These cells bear microvilli passing through the inner layer of the tunic and extending towards the apex of the papilla. The second cell type consists of elongated collocytes, rich in vesicles filled with a clear substance, likely mucus, displaced in the peripheral portion of the papilla surrounding the central fusiform cells. Cells of a third type are ciliated neurons localized in marginal position of the papillary body [31] (figure 1*i*).

An exception to the general rule of combining sensation and secretion in protruding papillae is found in larvae of the *Botrylloides* genus. Grave [34] described their papillae to contain sensory neurons but lacking secretory cells and proposed that the adhesion in *Botrylloides* is achieved by a suction-like mechanism, with the region surrounded by the palps acting as a sucker. However, Torrence & Cloney [33] reported that this region had sticky activity when probed with a needle. Each papilla of the *Botrylloides leachi* larva is formed by club-shaped cells, all supposed to be neurons (figure 1*j*). Two different types of neurons are present: the central neurons can be labelled with anti β-tubulin antibody and possess elongated sensory-like projections reaching the apex of the papilla, while the peripheral β-tubulin positive neurons contain serotonin in their distal endings but do not reach the apex of the papilla and thus form a ring at one-third of its length [32].

Indeed, no secreting cells are present in these papillar protrusions. Secreting-like cells are rather positioned in the centre of the larval head surrounded by the three papillae with histological features strikingly different from the surrounding cuboidal cells; they are elongated, have basal nuclei, are rich of secretory vesicles, pass through the inner layer of the tunic and form a sort of small glandular mucus-secreting organ [32] (figure 1*j*). Thus, it was confirmed that the papillae of this species have only sensory function and, in agreement with Grave [34], they could correctly be named sensory papillae to distinguish them from the glandular or adhesive papillae of other species. As a consequence, the sucker-like mechanism proposed by Grave [34] to explain the attachment of this larva, should be reconsidered: an anatomical conformation of the anterior region, forming a cup-like cavity, may contribute to larval attachment by creating a vacuum, with definitive adhesion being achieved by mucus secreted from the central region.

Thus, by comparing the cellular composition in the presented species, it is possible to draw a unifying picture where the cell types are always the same but their distribution is highly variable. In particular, in all analysed species, there are two types of neurons, the more exposed ‘higher’ neurons, and a second type of neuron with less exposed sensory terminals thus called ‘lower’ neurons.

The presence of two types of nervous cells in the papillae could be explained by taking into account that, in addition to their secreting function, the papillae of ascidian larvae also perform a mechanosensory function to trigger metamorphosis. It has been proposed that the two types of neurons are required to fulfil these different tasks [31].

Notably, the central neurons with terminal microvillus endings protruding beyond the apex of the papilla are the first to come into contact with the substrate during the exploratory period of the larva. Thus, it is reasonable to hypothesize that they may have a mechanosensory and/or chemosensory function. Instead, the terminal endings of lower neurons do not contact the substrate during the series of quick touches the larva uses to test the substrate. It is only after firm attachment of the larva by means of glandular organs that the papillae are pushed against the substrate to permit stimulation of the peripheral neurons.

Consequently, it is possible that, when the adhesion becomes permanent, these neurons are stimulated and participate in the signalling cascade that triggers metamorphosis, possibly by releasing a signalling molecule. One putative signalling molecule is the monoamine serotonin, which triggers metamorphosis in the larvae of the solitary ascidian *Phallusia mammillata* [16].

Collocytes, recognizable by their many vesicles are not always present in the body palps, such as in *B. leachi*. In all cases, however, secreting cells are present in the anterior ectoderm. It could be hypothesized that sensory neurons and collocytes were, originally, dispersed in the anterior ectoderm. All of the anterior ectoderm may have derived from an anterior placode [35] that formed an adhesive/sensory organ-like region. During evolution, the distribution of these cells may have become more organized with the formation of specialized sensory–secretory organs, the palps. Some palps included secreting cells in their bodies, in other cases secreting cells were clustered in the middle of the anterior ectoderm to form a secreting organ, leaving the palp with sensory function only.

Taken together, ascidian adhesive cells (collocytes) are always in the vicinity of two types of neurons, one resembling a primary sensory neuron and the other a mechano/chemoreceptor neuron. These three clearly distinguishable cell types arise from a common embryonic region and together form a functional unit to orchestrate the correct larval attachment. Further investigations will reveal more properties including specific secretory contents or contractile elements, at present not sufficiently analysed.

## 3. Larval adhesion: an evolutionary snap-shot of ancient building blocks?

It could be expected that the diversity apparent among marine animals would produce a large number of different adhesive mechanisms. On the other hand, evolutionary diversification seems to rely on the variation and recombination of existing structures or small functional units, like cell types or small regulatory networks. Such modular blocks are laid down during embryonic development and precursors for functionally similar structures may contain and combine information from evolutionary older cell types partially encoded by the geometric arrangements in precursor fields. Thus, a combination of innate cell properties and their geometric history is important. These functional building blocks are often conserved, between related species and even in more distant animal phyla. This is consistent with the idea of a comparative analysis between ascidian species and subsequently with less related animals, to extract functional signatures for both the ascidian palps and more commonly for adhesive organs of other animals.

Ascidian larvae, indeed, represent a transitory developmental stage that produces structures not persisting into adulthood, their sticky palps included. Two very interesting considerations with respect to finding general and specific adhesive organ building blocks should be taken into account. Firstly, larvae often resemble each other and may be an obligatory stage to go through for many related animals, likely representing an evolutionary stable body concept from which diversification was most successful. Larvae, thus, resemble an ‘evolutionary snap-shot’ containing ancient features shared by a group. This argument, which has spawned the popular phrase ‘ontogeny recapitulates phylogeny’, infers that an understanding of common phases of larval development gives an insight into evolutionary history. In this way, common components of larval development can be considered as building blocks that contain the common information for subsequent diversification. We will therefore present how the ascidian evolutionary snap-shot stage may be used to extract meaningful adhesive signatures.

Ascidians belong to the larger group of tunicates (also called urochordates), a sub-group of the chordates. It was their larvae that revealed them as close relatives to the vertebrates (including man), in that they possess an axial stabilizing rod of cellular composition (the notochord or chorda dorsalis), which resembles the embryonic spine primordium of vertebrates [36]. Sessile adults, in contrast, are morphologically very different forming specialized filter feeders covered with a protective cellulose like exoskeleton, the tunic.

Embryonic amenability in ascidians, notably a fixed cellular lineage, was recognized early on as predictive of the cell fates of individual cells [37]. More recently, the invariable lineage, combined with few and rather large cells, became the basis for advanced analyses of developmental and cellular processes, with partially similar strategies to the nematode *C. elegans* model system*,* but in a chordate context. This also led the solitary ascidian *C. intestinalis* to be among the first marine chordates to have their genome sequenced [38], and subsequently become an advanced molecular developmental model organism. Subsequently, molecular evidence clarified that within chordates (including cephalochordates, e.g. Amphioxus) tunicates turn out to be the sister group to the vertebrates [39].

The unique position of tunicates at the interface between invertebrates and vertebrates has, indeed, been subject to lively and ongoing debate to distinguish features in ascidians that possibly developed in a common ancestor from which both tunicates and vertebrates may have emerged. Much of the debate recently focused on finding aspects of typical vertebrate character in invertebrate chordates (ascidian larvae and Amphioxus, mostly) such as head sensory organs of placodal origin or of neural crest cells and their derivatives. In vertebrates, neural crest cells migrate out from the neural plate border and give rise to many cell types including the peripheral nervous system and pigmented cells. Migrating pigmented, HNK1-positive (a chick neural crest epitope) cells were identified in ascidians [40]. However, the second requirement to clearly identify these cells as neural crest derivatives is their developmental origin from the neural border. This was not confirmed as the cells turned out to be of mesodermal origin [40]. Interestingly, however, it was shown that precursors of the pigmented *Ciona* ocellus, that originate from the lateral neural plate border can migrate like neural crest if endowed with ‘migratory’ *twist* gene expression [41]. This finding suggests that ascidian ocellus precursors have a predisposed character of vertebrate neural crest, the latter having co-opted migratory properties into their gene regulatory repertoire.

Similar mechanisms of predisposed character might apply for the emergence of head sensory epithelia, which in vertebrates arise from placodes (ectodermal thickenings flanking the anterior neural plate border). Indeed, it has been suggested that ascidian head sensory epithelia (that include the palps) might form from ectodermal thickenings resembling ancestral placodes [35,42].

Thus, when considering sensory adhesive organs, the predisposition idea above suggests that precursors for functionally similar structures (sensory epithelia) may contain elements of evolutionary older cell types, combining cellular properties (secretion and neurosensation) with information about geometric precursor arrangements during embryology (common anterior epithelial precursors).

Cell type signatures can be distinguished by their ultrastructure. Sensory cells contain cilia in typical numbers and arrangements, collocytes contain vesicles, etc. These structures are normally made of variously modified protein products, visualized by specific staining techniques, including antibodies (figure 1). The cell type-specific protein repertoire is produced by cell type-specific expression of genes, called the transcriptome, representing the pool of protein-coding mRNAs. Large efforts are made to associate gene expression repertoires to cellular functions.

**Figure 2.**
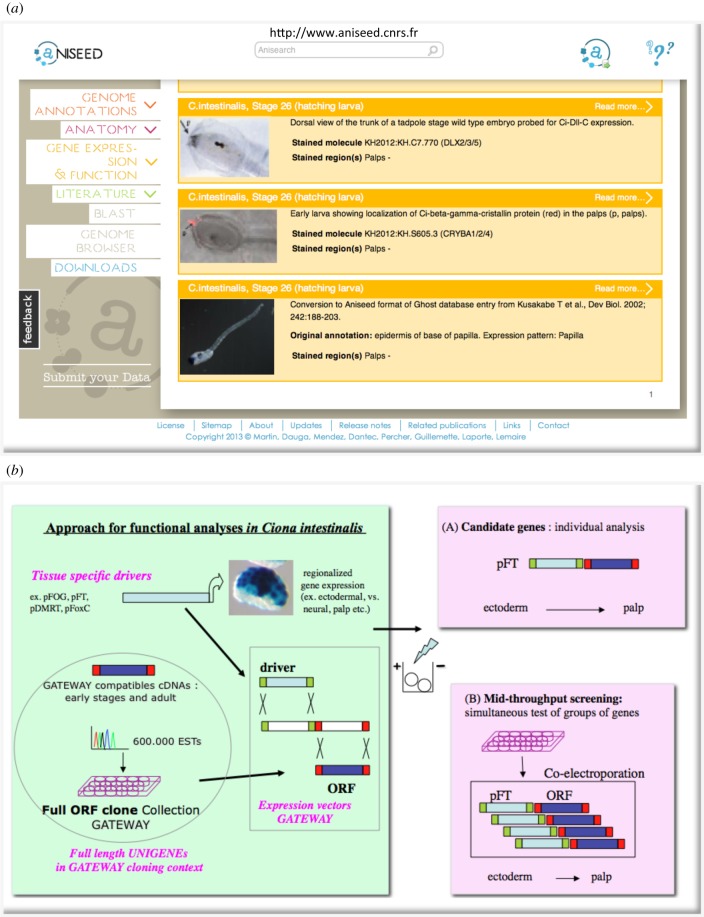
Functional genomics tools for ascidians. (*a*) ANISEED database. Screenshot of the gene expression interface, depicting palp expression of three different genes at the hatching larva (stage 25) of *C. intestinalis*. (*b*) Transient transgenesis by electroporation, available tools and approach. Tissue-specific drivers are regulatory regions of genes expressed in restricted regions of the embryo. They can be used to either drive reporter genes (LacZ in blue) for the analysis of upstream regulatory signatures or can be recombined with coding regions (ORFs) of candidate genes for the analysis of individual candidate genes (*a*), or for simultaneous testing of small groups of genes (*b*). Overexpression in palps precursors (in ectoderm, using pFOG, pFT or anterior ectoderm using pDMRT, pFoxC) could give insights into the contribution of the tested genes in adhesive organ formation. The GATEWAY cloning context (Invitrogen) was used for generating a vector suite [44] that allows efficient recombination of drivers and full ORF cDNA clones from arrayed genome wide clone collections [45].

In ascidians, genome and transcriptome sequencing (including EST collections) has been combined with invariant cellular lineage information, giving rise to vast expression catalogues that, in whole animals, correlate gene expression to individual or groups of cells [43]. Such repositories can now be queried [13], for example for genes specifically expressed in palps, as depicted in figure 2*a*.

Gain and loss of function experiments for individual gene products can be performed by, respectively, microinjecting coding mRNAs or inactivating oligonucleotides (morpholinos), into embryonic cells. Moreover, efficient DNA electroporation of fertilized eggs allows for tissue-specific probing of individual genes or analysis of their regulatory regions, notably in the context of larval adhesion. Electroporation was developed further for application to ascidians via the incorporation of adapted vectors and full-length cDNA collections (figure 2*b*). Electroporation vectors now support efficient recombinatorial cloning (by integrating the GATEWAY cloning system, Invitrogen) and facilitate protein tagging, notably with fluorescent proteins like GFP/Venus to determine the subcellular localization of proteins *in vivo* [44]. cDNA collections containing full open-reading frames (*Ciona* full ORFs) in GATEWAY compatible cloning context [45] are now widely used in the community to screen for context-specific gene function [46,47] or regulatory signatures [48]. An electroporation approach was also used for probing the above described neural crest features of *Ciona* light sensory organ precursors, upon twist expression [41] or for defining the gene regulatory networks (GRNs) guiding palp patterning and morphology, described further below [49,50].

Overall, the combination of developmental and molecular approaches has given insights into a number of cellular and developmental processes and into gene regulatory information in ascidians (reviewed in [51,52]) and also has provided a first regulatory blueprint for a chordate embryo [53]. Collections of genotypic and phenotypic informations from several databases have been hyperlinked and crossreferenced into a single repository, the ANISEED database [13]. This database can be interrogated and explored *in silico*. Such repositories can obviously be used to further study the ascidian adhesive organs (figure 2*a*), notably concerning their molecular signatures.

## 4. Molecular signatures in sensory adhesive organs

**Figure 3.**
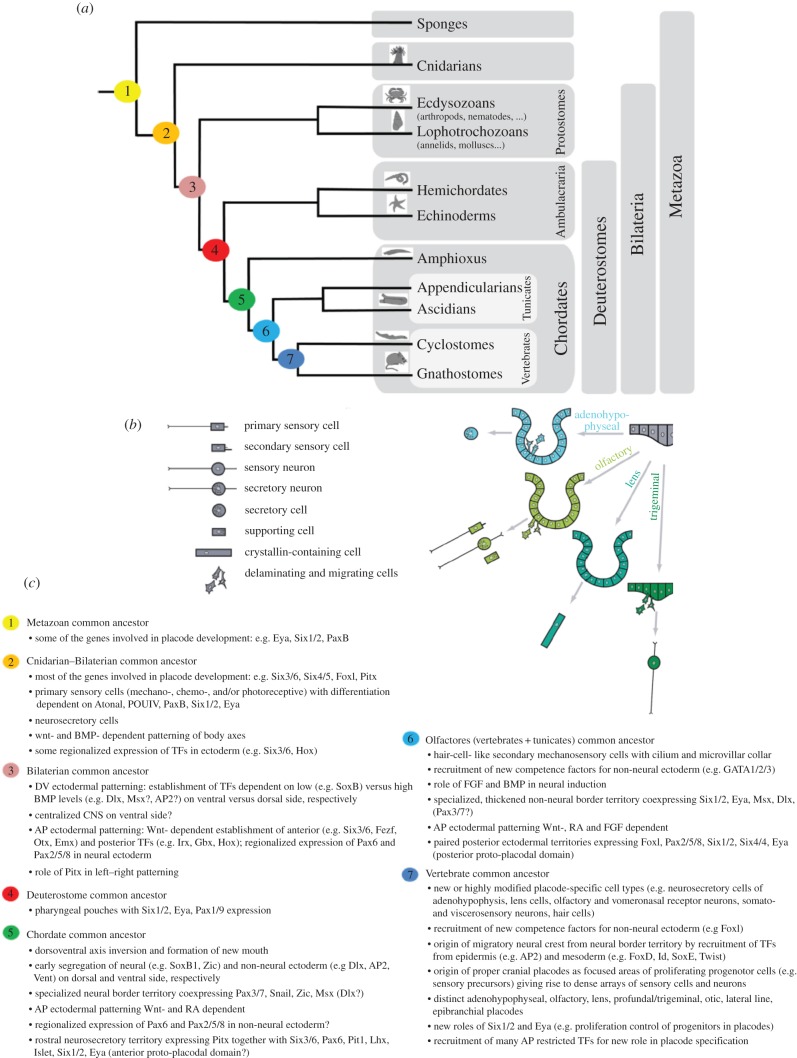
Evolution of sensory epithelia (that include ascidian palps) depicting cellular and molecular signatures (adapted from [54,55]). (*a*) Phylogenetic tree of metazoans depicting today's animal groups that contributed knowledge about cell types or similar regulatory mechanisms (transciption factors or signalling molecules). Numbers are positions of putative common ancestors (see (*c*)) with traits that likely have evolved to distinct characters in today's species (on the right). (*b*) The various cell types (left side) found in vertebrate sensory epithelia, are formed from separate embryonic regions, called placodes. The anterior placodes only are shown (right side) thought to have formed from a common territory in the chordate ancestor. (*c*) Cellular and molecular signatures are summarized that can be traced back to putative common ancestors on the tree of life.

Ascidian larval attachment organs, similar to the evolving neural crest discussed above, likely combine ancient characters, including primary sensory neurons, with novel features that may have further evolved to novel sensory organ structures in the vertebrates. Indeed, ascidian palps resemble vertebrate placode derivatives in relation to both their sensory function and their ectodermal origin near the border to the neural plate [35,54,55]. Resemblance to both invertebrate sensory structures, like the apical organs of many invertebrate larvae, and vertebrate placodes can be considered. Apical organs of many invertebrate larvae are typical transitory structures that allow them to sense the substrate, adhere to it by means of sticky mucus secretion and settle for metamorphosis that resorbs the adhesive organ and builds the adult tissues. They also contain flask-shaped neuronal cell types that are serotonin positive [56]. By contrast, vertebrate placodes give rise to diversified sensory structures in the new head of vertebrates arisen at the epidermal–neural interface and specialized on perceiving different types of sensations (olfaction, light, sound and pressure) (reviewed in [54]; figure 3). Although vertebrate placodes occupy distinct territories in the vertebrate larval stages, they are thought to have possibly emerged in ancestral protochordates from two larger, less specialized, anterior and posterior proto-placodal regions (as also mentioned in the first section). Figure 3*a* gives an overview on the phylogenetic positions of ascidians within the metazoan tree of life and depicts the various cell types generally found in ectoderm derived sensory structures in vertebrates (figure 3*b*), notably depicted according to their diversified presence in the anterior vertebrate placodes that are thought to have most features in common with ascidian palps [35].

To find molecular signatures of sensory adhesive organs, several transcriptome and gene regulatory features may be taken into account. These were recently summarized in a fairly complete and comparative way for sensory epithelia, found in the entire animal kingdom [54,55]. A comparative summary of cellular and molecular signatures for developing sensory epithelia is listed in figure 3*c*.

It is now well established that groups of functionally related genes are co-regulated by a few transcription factors (TFs), which are highly conserved during evolution. This guarantees synchronous gene expression needed for specific functionality of cells, such as neurons. Such arrangement in specific GRNs can reveal a molecular signature of cell types [57].

Secondly, the combination of several such signatures provides the mature cell with its specific subtype function, combining selective sets of terminal differentiation genes [58], for example in mechanosensory versus chemosensory neurons.

Thirdly, as discussed earlier, embryonic fields, from which groups of precursor cells arise, need to be taken into account. Signalling molecules and Hox genes provide animals with a coordinate system that allows cells to arise in different regions of the body. Such signals for dorsal/ventral (D/V), anterior/posterior (A/P) or left/right (L/R) repetitively define and redefine positional information along the body axes while the embryo grows.

Finally, the diversity of today's species is likely generated from ancestral repertories of such conserved small GRNs. Diversification can arise in two ways: through loss of certain properties by division of labour [57] or through novel acquisition of sets of genes by co-option [59]. By division of labour, an ancestral cell, for example a secretory sensory neuron, can give rise to different cell types, such as sensory neurons or secretory cells. Through co-option, as depicted earlier, neural crest may have gained migratory properties through acquisition of the twist downstream regulatory network.

Likely relevant for sensory adhesive organ formation, ascidian palps included, is an ancient TF signature for primary sensory neuron precursors: Eya, Six1/2, PaxB, POUIV and Atonal. This signature seems to have persisted since the beginning of multicellular life. Another ancient signature is that for axis formation, by Wnt (and BMP), RA and Wnt/FGF for A/P patterning and by FGF and BMP signalling for neuro-ectodermal patterning. Wnt-dependent A/P signatures include anterior TFs Otx, Six3/6, (Emx), versus posterior TFs Gbx and Hox1.

Conserved signatures for neural versus epidermal character often contain TFs Zic and SoxB1 versus Dlx and AP2, in respective precursors. Neural border territories are defined by Msx in combination with Zic and Dlx, and for Pax genes, a specialization for neural (Pax3/7) and non-neural ectoderm (Pax6 and Pax2/5/8) seems a common theme. Various combinations of such elements can then be observed in specialized territories, for example an anterior proto-placodal domain may be set up in chordates by the combined expression of Otx, Eya, Six1/2, Six3/6 and Pax6 versus a posterior proto-placodal domain by combinations of Eya, Six1/2, Pax 2/5/8, Six4/5 and FoxI.

In vertebrates, such larger sensorial territories are then further evolved to subdivided and specialized domains, along the ‘division of labour concept’ with additional specialized TFs for each of the placodal sub-domains. New cellular properties have been achieved by the recruitment of conserved signatures, such as the twist regulatory networks in vertebrate neural crest for migratory properties of cells. These TFs will coordinate the timely and positionally correct production of terminal differentiation genes found in the functional organ. A mixture and subsets of the above are likely found in differential transcriptomes.

In light of such ancient signatures, we will now briefly revisit some aspects of molecular knowledge in ascidians, relevant to palp formation. The fast and simple development can be seen as an advantage to extract minimally required genetic inputs. With large cells containing fixed lineage determinants, ascidians rely less on coordinating morphogen gradients, but rather use a few local interactions with neighbouring domains to specify intermediate cell fate, such as the neural/epidermal interface from which the palps arise. Indeed, both the ascidian palps (with neural and secretory cell types) and the larval brain arise from precursors of the fixed anterior neural lineage. This region arises early on after simple subpartitioning of the embryo (reviewed in [51]), when vegetal (mesendodermal) cells first accumulate β-catenin and oppose animal ectodermal GATA4/5/6 TF activity [47]. The β-catenin target FGF9/16/20 then signals from the vegetal territory to locally trigger the pre-neural, Otx marked, state through Ets and GATA4/5/6 factor synergism [60] but only in selected ectodermal cells that show largest cell surface contact to neighbouring FGF cells [61]. Subsequently, the Otx positive neural state is maintained only in anterior palp/brain precursors, also expressing DMRT [43]. One division later, the neural/non-neural fate is segregated further, again by FGF signal switching. FoxC expression in the future palp territories (row V and VI cells of the neuro-ectodermal plate) is made possible by repression of FGF signalling, while adjacent, neural fated cells (row III and IV) express ZicL [49]. Robustness is added through FoxC repressing ZicL, and an independent mechanism for delaying the neural ZicL expression [62]. Thereafter, FoxC activates Six1/2 in the palp territory, followed by Eya, Emx and the neural differentiation markers COE and POUIV [42,46,63]. Dll-C then appears in palp precursors where it persists to late larval stages (figure 2*a*). Interestingly, Dll-C is also expressed, transiently, in the ‘neurogenic’ epidermis midline where it depends on Msx and Admp [43]. Midline expression of Dll-C disappears earlier than in palps, after epidermal sensory neurons have formed by Notch lateral inhibition [46]. Finally, βγ-crystallin, typically found in vertebrate lens fibre cells, is expressed in both palps (figure 2*a*) and the otolith of the larval brain, with its regulatory region being able to drive lens expression in the frog [64]. Common chordate regulators for βγ-crystallin expression, however, remain to be identified [65]. Overall, several of the above-mentioned ancient signatures for both cell type and patterning along the body can be found in developing ascidian larvae. Notably, primary sensory neurons, expressing an ancient neural signature, including POUIV and COE, occur in different regions of the body (palps or epidermis midline) likely by co-option of a coordinating TF, possibly Dll-C. βγ-Crystallin may fulfil an ancient structural role. Very recent indications for an ancient cell morphology signature come from palps in ascidians. Here, Islet TF causes the columnar cell shape change in the palp precursors with circular Emx expression delimiting the three protrusions [50]. Interestingly, βγ-crystallin is downstream of Islet in palps and both are co-expressed in the otolith.

## 5. Conclusion

Comparative analysis of structural and cellular components of ascidian adhesive organs reveals a common cellular signature and a likely sequential suite for larval adhesion. Many questions remain open about both cellular and mechanistic properties of cells involved, notably contents and functioning of collocytes likely triggered for secretion by mechano/chemosensory stimuli. A comparative transcriptomic approach of specific tissues or organs, such as for sensory adhesive structures of marine animals, will reveal batteries of genes specifically expressed in these tissues. Combining proteomics and transcriptomics [66] may reveal strong candidates to participate in the building of the adhesive organs and its products. Targeted gene interference approaches such as gene knockdown and efficient overexpression, possible in *Ciona*, will be instrumental in probing the involvement of individual genes in the functioning of the adhesive organ. This can best be tested during the dynamic process of tissue formation, either in regenerating or newly developing tissues in embryos. Ascidians may be particularly well suited for such approach as they form adhesive organs in their larvae that can be dynamically and comparatively observed, both for their cellular composition as depicted in the first part of this review and their molecular repertoires described in the second part of this review. Studies in several model organisms from scattered and representative animal phyla tell us about initial cellular and molecular signatures that can be taken into consideration. A comparative analysis has taught us about the hierarchic and modular construction of tissues and organs, with molecular signatures for both the cell types and their location within organisms, as depicted for sensory epithelia. As some of these combinatorial codes are highly conserved in evolution it is very likely that phenotypically similar cells or similar sensory organs may use, at least in part, similar building blocks to synchronize gene repertoires. Such consideration will aid in the identification of adhesive signatures, at a cellular and molecular level. In addition to the relevance of such studies to the understanding of fundamental biological processes, deep knowledge of the adhesive strategies of ascidians will be instrumental for the biomimetic design of ascidian-specific antifouling solutions and of novel physiologic glues.
